# Metagenomic sequencing characterizes a wide diversity of viruses in field mosquito samples in Nigeria

**DOI:** 10.1038/s41598-022-11797-2

**Published:** 2022-05-10

**Authors:** Judith U. Oguzie, Udoka C. Nwangwu, Paul E. Oluniyi, Testimony J. Olumade, Uwem E. George, Akano Kazeem, Bolajoko E. Bankole, Farida O. Brimmo, Chukwuemeka C. Asadu, Okechukwu C. Chukwuekezie, Josephine C. Ochu, Catherine O. Makwe, Festus A. Dogunro, Cosmas O. Onwude, William E. Nwachukwu, Ebuka K. Ezihe, Gilkenny K. Okonkwo, Ndubuisi E. Umazi, Jacob Maikere, Nneka O. Agashi, Emelda I. Eloy, Stephen O. Anokwu, Angela I. Okoronkwo, Ebuka M. Nwosu, Sandra O. Etiki, Ifeoma M. Ngwu, Chikwe Ihekweazu, Onikepe A. Folarin, Isaac O. O. Komolafe, Christian T. Happi

**Affiliations:** 1 African Centre of Excellence for Genomics of Infectious Diseases (ACEGID), Redeemer’s University, Ede, Osun Nigeria; 2 Department of Biological Sciences, Faculty of Natural Sciences, Redeemer’s University, Ede, Osun Nigeria; 3 National Arbovirus and Vectors Research Centre (NAVRC), Enugu, Enugu Nigeria; 4 Médecins Sans Frontières (MSF Belgium), Bruxelles, Belgium; 5 Nigeria Center for Disease Control, Abuja, Nigeria

**Keywords:** Computational biology and bioinformatics, Molecular biology

## Abstract

Mosquito vectors are a tremendous public health threat. One in six diseases worldwide is vector-borne transmitted mainly by mosquitoes. In the last couple of years, there have been active Yellow fever virus (YFV) outbreaks in many settings in Nigeria, and nationwide, entomological surveillance has been a significant effort geared towards understanding these outbreaks. In this study, we used a metagenomic sequencing approach to characterize viruses present in vector samples collected during various outbreaks of Yellow fever (YF) in Nigeria between 2017 and 2020. Mosquito samples were grouped into pools of 1 to 50 mosquitoes, each based on species, sex and location. Twenty-five pools of *Aedes *spp and one pool of *Anopheles *spp collected from nine states were sequenced and metagenomic analysis was carried out. We identified a wide diversity of viruses belonging to various families in this sample set. Seven different viruses detected included: Fako virus, Phasi Charoen-like virus, Verdadero virus, Chaq like-virus, Aedes aegypti totivirus, cell fusing agent virus and Tesano Aedes virus. Although there are no reports of these viruses being pathogenic, they are an understudied group in the same families and closely related to known pathogenic arboviruses. Our study highlights the power of next generation sequencing in identifying Insect specific viruses (ISVs), and provide insight into mosquito vectors virome in Nigeria.

## Introduction

One in six diseases worldwide is transmitted by mosquito vectors^1^. It is estimated that half of the world's population is in danger of viral illnesses spread by mosquitoes and causing death in millions of people annually. Rift Valley fever virus (RVF), Dengue virus (DENV), Zika virus (ZIKV), Chikungunya virus (CHIKV), Yellow fever virus (YFV), Japanese encephalitis virus (JEV), and Ross River virus (RRV) are just a few of the mosquito-borne viruses that have caused disease epidemics both in humans and animals. Additionally, an increasing number of viruses specific to arthropods, classified as insect-specific viruses (ISVs), have been identified in the last two decades in diverse mosquito populations around the world^2–8^. ISVs and pathogenic arboviruses evolutionary relationship remains uncertain^9^. However, evolution from arthropod-specific viruses has been assumed for the genus Flavivirus. Pathogenic viruses are thought to have evolved from insect-specific to dual host viruses^10–12^.

Metagenomic sequencing has increased exponentially the number of mosquito-borne viruses isolated in the last couple of years and further provided fresh insight into the enormous complexity and variety of invertebrate RNA viruses^4,6,11^. Recently, in Nigeria, metagenomic sequencing was used to identify an ongoing yellow fever outbreak and its aetiology and inform real-time public health actions, resulting in accurate and timely disease management and control^13^. A greater understanding of the virome in mosquito species in Nigeria could allow for a more accurate assessment of mosquito-borne disease risk, vector competence and mosquito management.

In the course of various YFV outbreaks in Nigeria between 2017 and 2020, we collected vector samples (mostly *Aedes* spp) in sites where there were active YFV cases. Next-generation sequencing (NGS) was carried out on 26 pools of 1300 mosquitoes (50 mosquitoes per pool) across nine (9) states in Nigeria using a metagenomic protocol as previously described^14^. In this paper we present our findings and discuss the implications.

## Results

### Metagenomics analysis

**Figure 1:**
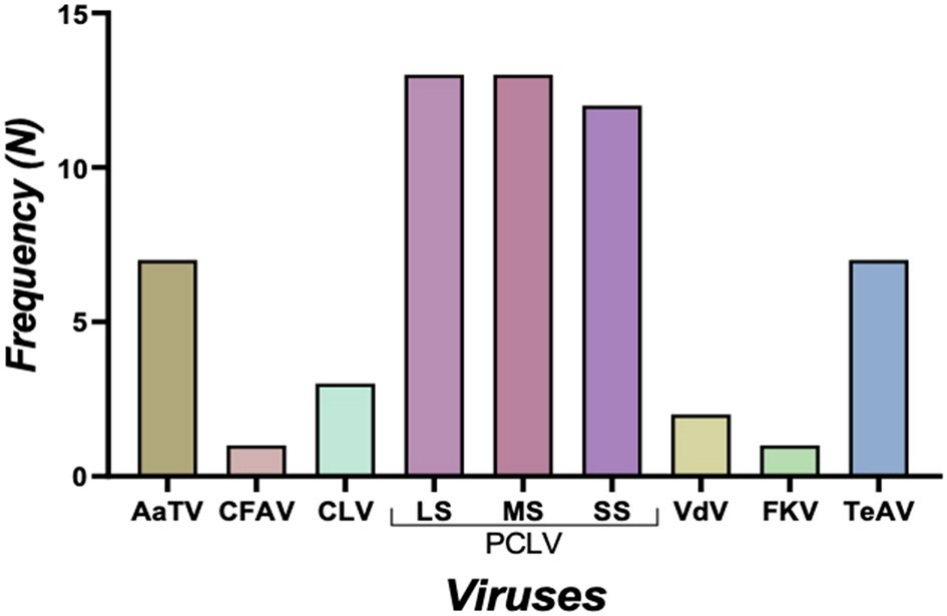
Frequency distribution of the viruses found in the mosquito pools. *AaTV* *Aedes aegypti* totivirus, *CFAV* cell fusing agent virus, *CLV* Chaq-like virus, *PCLV* Phasi Charoen-like phasivirus (L segment, M segment, and S segment), *VdV* Verdadero virus, *FKV* Fako virus, *TeAV* Tesano Aedes virus.

Metagenomics analysis carried out on sequenced samples revealed the presence of a wide range of viruses (Fig. 1). The following Families of viruses; *Bunyavirales* (Phasi Charoen-like phasivirus), *Iflaviridae* (Tesano Aedes virus), *Partitiviridae* (Chaq-like virus and Verdadero virus), *Flaviviridae* (Cell fusing agent virus) and *Totiviridae* (*Aedes aegypti* totivirus (AaTV) were detected.

The mosquito species that were pooled after morphological identification included the following: *Aedes aegypti* (n = 12 pools), *Aedes albopictus* (n = 10 pools), *Aedes simpsoni complex* (n = 2) *Aedes luteocephalus* and *Anopheles coustani* (n = 1 pool each) with *Aedes aegypti* and *Aedes albopictu*s accounting for more than 80% of the mosquito pools analyzed. Of the 26 pools, irrespective of the mosquito species, the number of pools positive for *Aedes aegypti* totivirus (AaTV), cell fusing agent virus (CFAV), Chaq like-virus (CLV), Phasi Charoen like phasivirus (PCLV) (L segment), PCLV (M segment), PCLV (S segment), Verdadero virus (VdV), Fako virus (FKV) and Tesano Aedes virus (TeAV), were 7, 1, 3, 13, 13, 12, 2, 1 and 7 respectively (Fig. 1).

The prevalence of PCLV was significantly higher compared to other viruses (P < 0.0001). Table 1 shows the distribution of the virus in the mosquito pools. The distributions were estimated as the proportion of the positive pool over the total pool analyzed for each pathogen. It is an expression used to indicate the proportion/ratio/frequency in various cohorts in a population. Aedes aegypti and Aedes albopictus were the most common mosquitoes in the study areas. They accounted for > 80% of the pools analyzed in the study. In addition, no viral genome was assembled from Aedes simpsoni, Aedes simpsoni complex and Anopheles coustani pools. Excluding these minor groups of the mosquito pools, distributions of the viruses between the two major species were similar. In *Aedes aegypti*: Excluding CFAV from statistical comparison, the prevalence of PCLV was significantly higher compared with other viruses (P = 0.006; Table 1). In *Aedes albopictus*: Excluding FKV and TeAV from statistical comparison, the prevalence of the viruses was similar (P = 0.41; Table 1). FKV and TeAV were excluded because the proportions were zero, and there will be no meaningful statistical comparison with zero value (e.g. 0 of 10).

**Table 1:** Distribution of the viruses based on the species of mosquitoes.

Mosquito species	Viruses									P value
	AaTV	CFAV	CLV	PCLVLS	PCLVMS	PCLVSS	VdV	FKV	TeAV	
*Aedes aegypti*	4/12	0/12	2/12	9/12	9/12	9/12	0/12	2/12	1/12	0.003*
*Aedes albopictus*	3/10	1/10	1/10	4/10	4/10	3/10	2/10	0/10	0/10	0.41**
*Aedes luteocephalus*	0/1	0/1	0/1	0/1	0/1	0/1	0/1	0/1	0/1	–
*Aedes simpsoni C*	0/2	0/2	0/2	0/2	0/2	0/2	0/2	0/2	0/2	–
*Anopheles coustani*	0/1	0/1	0/1	0/1	0/1	0/1	0/1	0/1	0/1	–

*AaTV* *Aedes aegypti* totivirus, *CFV* cell fusing agent virus, *CLV* Chaq-like virus, *PCLP* Phasi Charoen like phasivirus (L segment, M segment, and S segment), *VdV* Verdadero virus, *FV* Fako virus, *TeAV* Tesano Aedes virus, *C* complex.

*CFAV was excluded from this analysis.

**FKV and TeAV were excluded from this analysis.

### Genome assembly and phylogenetic analysis

Genome assembly of viruses detected by our metagenomics pipeline^15^ was attempted to characterize the diversity of these viruses and their evolutionary relationship with previously reported viruses. A heatmap showing the distribution of viruses is shown in Supplementary Fig. S1.

### Totiviridae

**Figure 2:**
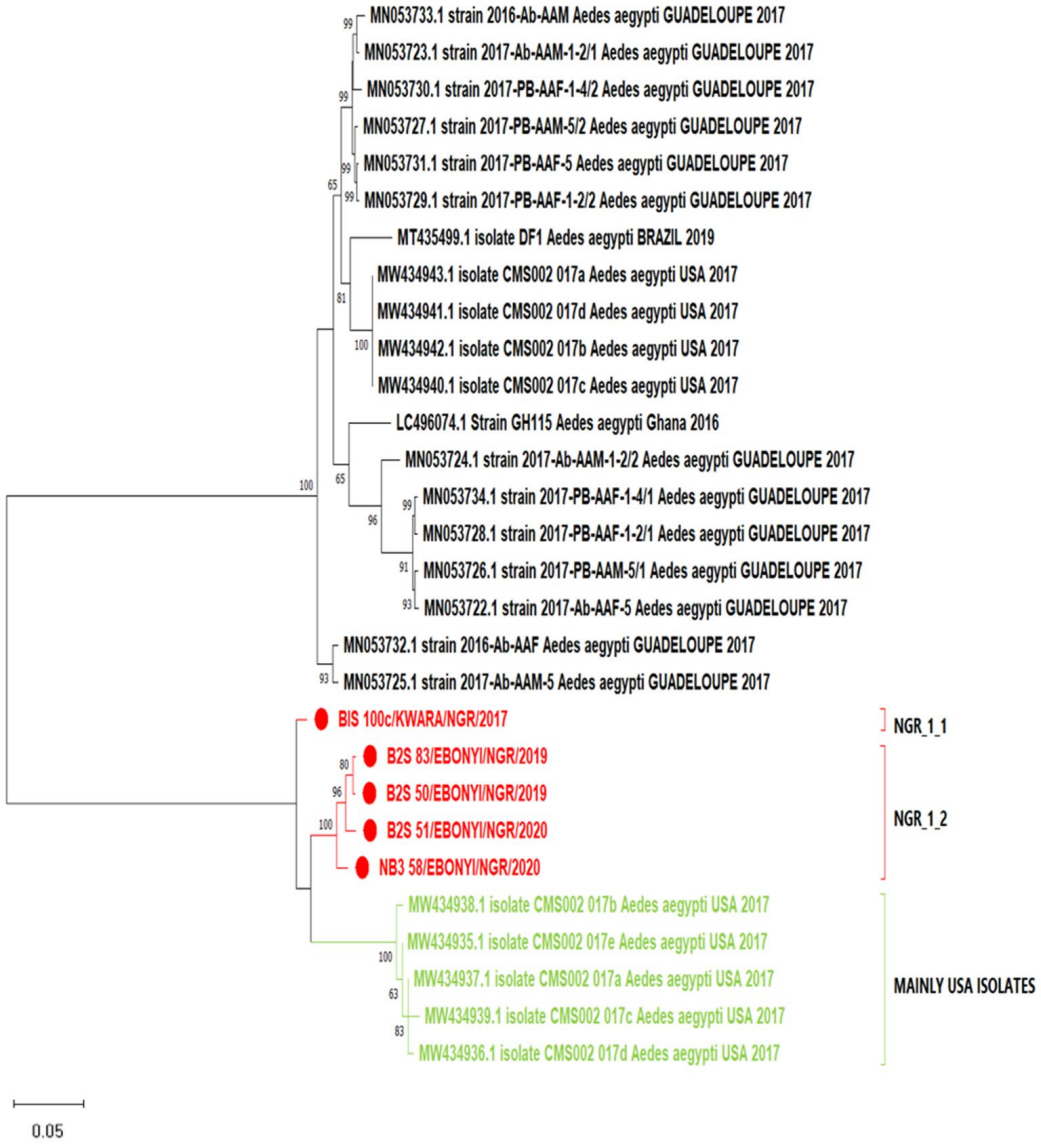
Phylogenetic analysis of orf1 nucleotide sequences of *Aedes aegypti* totivirus (AaTV). The evolutionary history was inferred using the Maximum Likelihood method and Tamura-Nei model with 1000 bootstrap replicates. The numbers at branch nodes indicate the bootstrap values ≥ 50%. All the reference strains are identified by name and GenBank accession number. Virus strains characterized in this study are highlighted in red.

We assembled six (five full and one partial) genomes for *Aedes aegypti* totivirus (AaTV) out of the seven pools with reads for this virus. Phylogenetic analysis revealed that the *Aedes aegypti* totivirus sequences from this study clustered closely together and fell in the same major clade. Within the major clade, the sequence from Kwara state (BIS 100c) branched out on its own while the Ebonyi sequences (NB3 58, B2S 51, B2S 50 and B2S 83) clustered together, which could imply a localized/within-state spread of the virus. The short branch lengths of the sequences also show the limited diversity of the virus in Nigeria (Fig. 2).

### Bunyavirales

**Figure 3:**
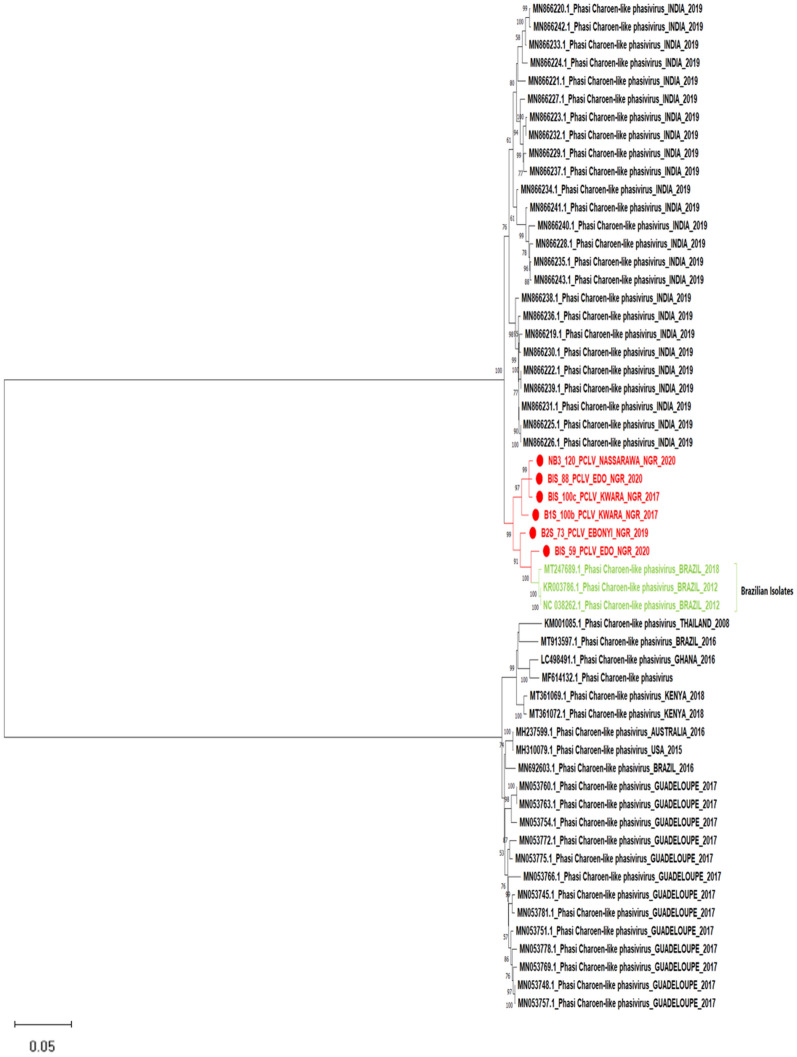
Maximum likelihood phylogenetic tree showing evolutionary relationship between the L segment (RdRp) of Phasi Charoen like phasivirus sequences from this study and those obtained from the NCBI database. The evolutionary history was inferred using the Maximum Likelihood method and Tamura-Nei model with 1000 bootstrap replicates. The numbers at branch nodes indicate the bootstrap values ≥ 50%.

**Figure 4:**
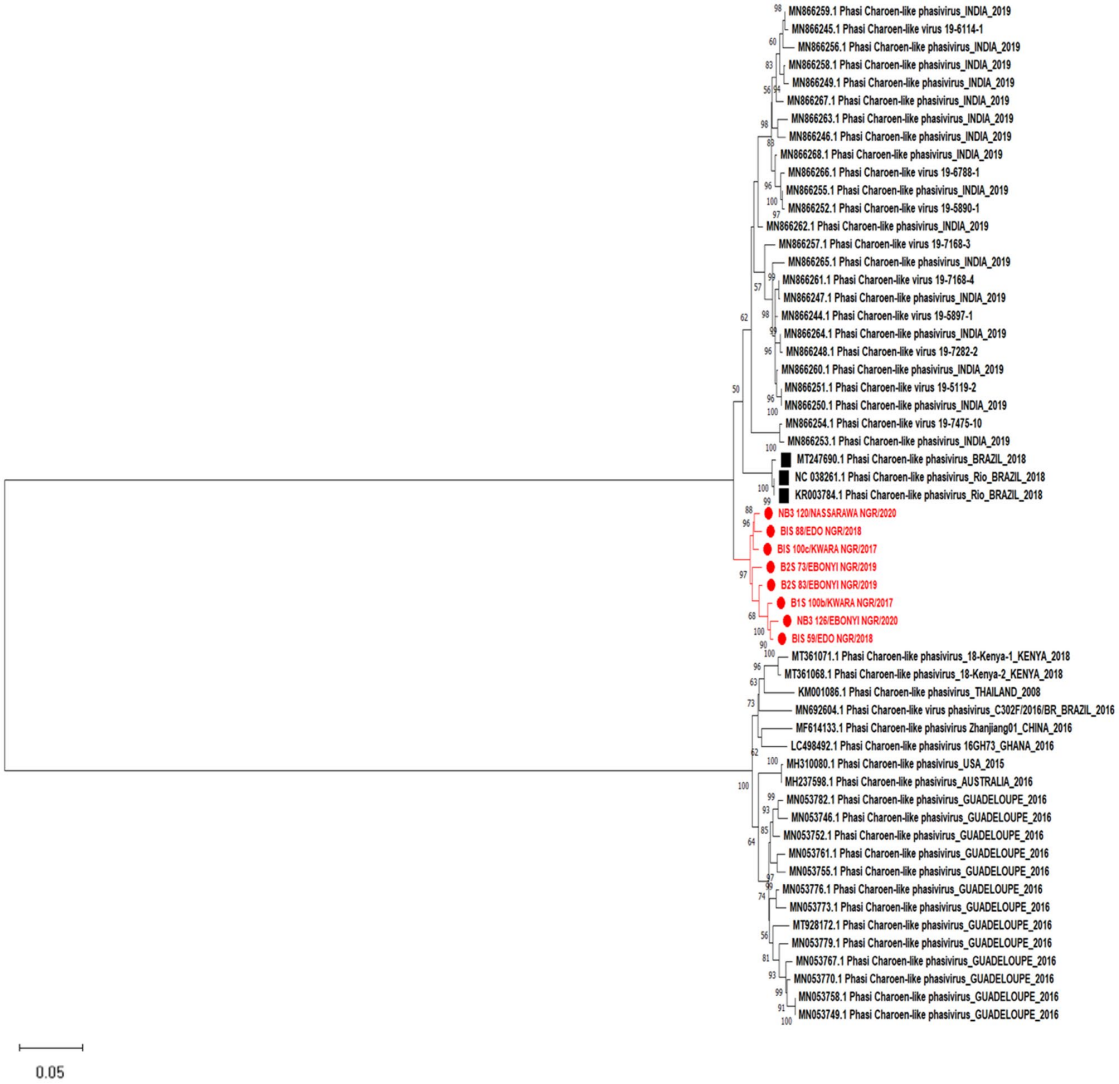
Maximum likelihood phylogenetic tree showing evolutionary relationship between the M segment (glycoprotein) of Phasi Charoen like phasivirus sequences from this study and those obtained from the NCBI database. The evolutionary history was inferred using the Maximum Likelihood method and Tamura-Nei model with 1000 bootstrap replicates. The numbers at branch nodes indicate the bootstrap values ≥ 50%.

**Figure 5:**
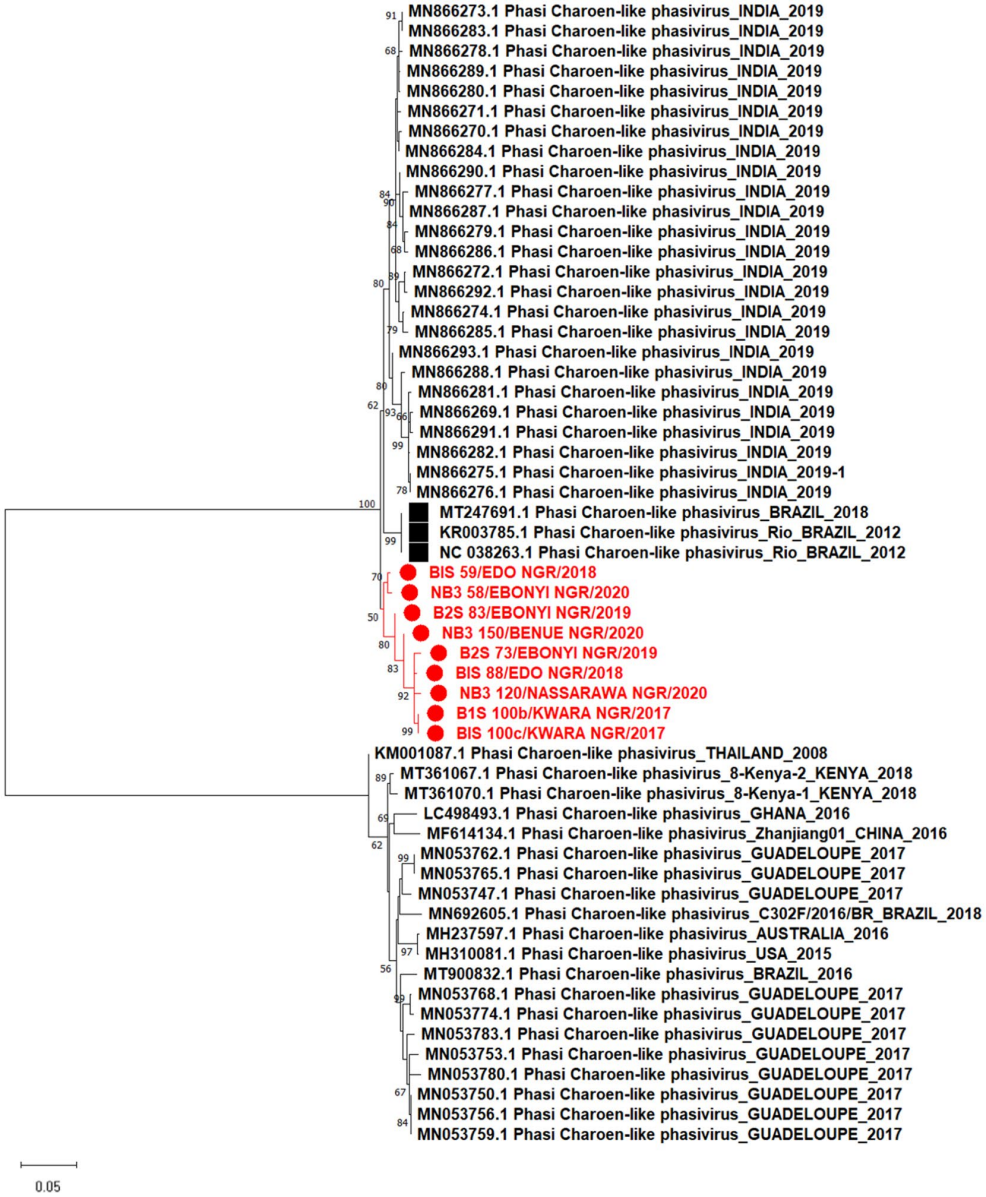
Maximum likelihood phylogenetic tree showing evolutionary relationship between the S segment (Nucleocapsid) of Phasi Charoen like phasivirus sequences from this study and those obtained from the NCBI database. The evolutionary history was inferred by using the Maximum Likelihood method and Tamura-Nei model with 1000 bootstrap replicates. The numbers at branch nodes indicate the bootstrap values ≥ 50%.

Phylogenetic analysis revealed similar clustering patterns across the three segments of the virus in Nigeria. According to states, there is no observed clustering pattern showing the virus's limited diversity in the country (Figs. 3, 4, 5).

Other viruses (Tesano Aedes Virus**,** Chaq-like virus, Fako virus and Verdadero virus) were assembled from our sequenced data. We did not construct phylogenetic trees due to the lack of substantial genomes on NCBI for proper comparison. In which cases, only one to three genomes are available in the database.

The percentage similarity for these viruses assembled from this study is detailed in Tables 2 and 4.

**Table 2:** Genome characterization of Fako virus genome described in this study.

Segment (accession no.)	Segment length (bp)	ORF length (aa)	Protein	Encoded gene	Closest strain in GenBank	Identity (%)
OM522898	2398	132	VP2	RNA-dependent RNA polymerase	KM978429.1	94.29
OM522899	2925	213	VP3	Major capsid protein	KM978432.1	98.22
OM522900	3176	528	VP4	Nonstructural protein	KM978433.1	97.95
OM522901	2983	286	VP5	Turret protein	KM978436.1	97.00

## Discussion

Several studies have applied metagenomics sequencing in isolating viruses from mosquitoes in Africa^16–19^. Our study reports the first metagenomics analysis of mosquitoes in Nigeria. Not only did we detect the presence of viral reads from the samples, but all the reads were also sufficient to assemble genome sequences.

We detected the presence of Fako virus, *Aedes aegypti* totivirus, Cell fusing agent, Tesano Aedes virus, Phasi Charoen-like phasivirus Chaq-like and Verdadero viruses, all of which are being reported for the first time in Nigeria. Chaq-like virus and Verdadero viruses reported in this study are the first reports in Africa.

Our study also highlights the application of next generation sequencing in identifying ISVs and granting insight into the mosquito virome in Nigeria.

*Aedes aegypti* and *Aedes albopictu*s were the most common mosquitoes in the study areas. They accounted for > 80% of the pools analyzed in the study. The reason is that our sampling was biased toward *Aedes species* as essential vectors of YFV. They are also the dominant members of their genus breeding around domestic and peri domestic areas of human habitations^20^.

We assembled four (segments 2,3,4 and 5) and a partial first segment out of the nine segments of the Fako virus; reovirus of the genus Dinovernavirus. The first report of the virus is in mosquito pools from Cameroun^16^. This virus is maintained via mosquito to mosquito transmission and might have evolved from its initial ancestor through loss of function activities. There is no report of human infection with FKV. Our genome was isolated from one *Aedes aegypt*i pool from Edo State. Across all segments, our sequences were 96.7% similar to the first sequence from Cameroun (Table 2).

There are seven genomes (four full and three partial) for Tesano Aedes virus (TeAV) in mosquito pools. TeAV is a member of the Iflaviridae family and was first isolated from Mosquito samples in Ghana^17^. The virus has shown evidence of vertical transmission^17^. It increases the growth of Dengue virus 1 (DENV-1) in a concentration-dependent manner under laboratory conditions^17^. Members of this family are known to infect insects with no reports of human infection. On average, our sequences were 96% similar to the only sequence for the TeAV available on NCBI (Table 4).

We had five complete genomes and one partial genome assembly for AaTV. This virus is a member of the *Totiviridae* family and a group of dsRNA viruses that infect fungi, protozoa, or invertebrates^21^. AaTV was highly distributed and present in mosquito pools from Kwara and Ebonyi States. Phylogenetic analysis of the virus genomes revealed that the Nigerian Strains clustered together in the same major clade, independent of the American/Asian/European lineages, implying continuous evolution and diversity of the virus (Fig. 2). Sequences obtained from the same state (Ebonyi State) clustered closely in a sub-clade on the tree, resulting from the localized spread of the virus among the mosquito vectors in this community.

In addition, we had one partial genome assembly of a flavivirus; Cell fusing agent virus. Many medically important arboviruses belong to the *Flaviviridae* family. Our sequence shares 97.8% identity with the sequence from Uganda. CFAV is the first ISV reported and named after its characteristic CPE of fusion of cells^22^. Two decades later, researchers sequenced CFAV in 1992^23^. The virus has been isolated from *Aedes* species in dengue endemic areas^6,24–28^. There is a close phylogenetic relationship between insect-specific (ISF) and medically important flaviviruses. This information could be valuable in understanding how ISFs enable/inhibit transmission of arboviruses in nature and their possible use as agents of biological control of vectors^2^. A study by Baidaliuk et al., 2019 evaluated how CFAV affects ZIKV and DENV-1 in vitro and vivo^29^. Their findings showed a negative correlation both in-*vitro* and *in-vivo,* indicating a decrease in transmission in both viruses due to the presence of CFAV.

Furthermore, we assembled 13 genomes for the L and M segments, as well as 12 for the S segment of Phasi Charoen-like-phasivirus (PCLV)- a bunyavirus first isolated from the Phasi Charoen district of Thailand from wild-caught *Aedes aegypti* larvae^30^. The phylogenetic tree based on the S segment (Nucleocapsid) and M segment (glycoprotein) displayed these segments as being in a separate cluster independent of previously detected lineages (Figs. 3 and 4). However, RdRp sequences encoded by the L segment displayed the PCLV from our study as being in the same clade as RdRp of previously detected PCLV from *Aedes aegypt*i from Brazil in 2012 (Fig. 5). We have characterized our assemblies for the three segments of the virus (Table 3). There may be a link between PCLV and the transmission of arboviruses, e.g., Ae*. albopictus* cell line Aa23, persistently infected with CFAV, inhibited ZIKV replication and transmission^31^. At the same time, another study isolated PCLV from *Ae. aegypti* naturally infected with the Chikungunya virus (CHIKV)^32^ during an arbovirus surveillance program. The relationship between PCLV and CHIKV transmission is still unknown and needs further investigation.

**Table 3:** Genome characterization of Phasi Charoen-like phasivirus genome described in this study.

Segment	Strain name	Segment (accession_no)	Segment length (bp)	ORF length (aa)	Encoded gene	Closest strain in GenBank	Identity (%)
L	B1S_100b_PCLP	OM522847	6781	2217	RNA-dependent RNA polymerase	MT361069.1	97.50
	B2S_48_PCLP	OM522851	6156	279	RNA-dependent RNA polymerase	MN053766	98.44
	B2S_73_PCLP	OM522854	6471	351	RNA-dependent RNA polymerase	MT361069.1	97.62
	B2S_83_PCLP	OM522855	5906	316	RNA-dependent RNA polymerase	MT361069.1	95.93
	B1S_59_PCLP	OM522848	6731	2217	RNA-dependent RNA polymerase	MH237599.1	98.60
	B1S_88_PCLP	OM522849	6738	2217	RNA-dependent RNA polymerase	MT361069.1	97.58
	B1S_100c_PCLP	OM522856	6514	837	RNA-dependent RNA polymerase	MT361069.1	92.00
M	B1S_100b_PCLP	OM522860	3819	1237	Glycoprotein	MH237598.1	97.07
	B2S_48_PCLP	OM522870	3564	501	Glycoprotein	MN053776.1	97.41
	B2S_50_PCLP	OM522869	3346	192	Glycoprotein	MN053776.1	96.84
	B2S_73_PCLP	OM522871	3521	1492	Glycoprotein	MN053776.1	97.13
	B2S_83_PCLP	OM522868	3600	620	Glycoprotein	MN053782.1	95.72
	B1S_59_PCLP	OM522861	3768	1237	Glycoprotein	MH237598.1	96.97
	B1S_88_PCLP	OM522862	3833	1237	Glycoprotein	MH237598.1	94.04
	B1S_100c_PCLP	OM522867	3593	558	Glycoprotein	MH237598.1	97.84
	NB3_58_PCLP	OM522866	3354	217	Glycoprotein	MN053776.1	98.20
	NB3_120_PCLP	OM522863	3768	1237	Glycoprotein	MH237598.1	97.21
	NB3_126_PCLP	OM522872	3614	552	Glycoprotein	MN053776.1	96.77
	NB3_150_PCLP	OM522865	3620	350	Glycoprotein	MN053776.1	97.57
S	B1S_100b_PCLP	OM522873	1331	268	Nucleocapsid	MN866293.1	96.31
	B2S_73_PCLP	OM522876	769	250	Nucleocapsid	MN866293.1	95.84
	B2S_83_PCLP	OM522882	728	109	Nucleocapsid	MN866293.1	97.67
	B1S_59_PCLP	OM522877	1326	268	Nucleocapsid	MN866293.1	96.98
	B1S_88_PCLP	OM522878	1331	268	Nucleocapsid	MN866293.1	96.53
	B1S_100c_PCLP	OM522879	861	265	Nucleocapsid	MN866293.1	96.28
	NB3_58_PCLP	OM522884	1290	199	Nucleocapsid	MT361067.1	95.06
	NB3_120_PCLP	OM522880	1325	268	Nucleocapsid	MN866293.1	96.00
	NB3_126_PCLP	OM522881	665	189	Nucleocapsid	MT361067.1	98.05
	NB3_150_PCLP	OM522883	1024	117	Nucleocapsid	MN866293.1	96.62

Partitiviruses are known to infect a vast host, including plants, fungi with some members of this genus recently discovered in arthropods^8,33,34^. In this study, three (3) Chaq-like viruses were found from *Aedes *spp pools. The BLASTn search results of the three sequences from our study showed high nucleotide sequence identity (98.7%) to strain CLv.PozaRica20 (MT742176.1) was isolated from *Aedes aegypti* in Mexico in 2020. Showing the virus may be widely disseminated in the mosquito vector. Only three sequences are available on NCBI for this virus as of 23rd of October 2021, with limited information.

**Figure 6:**
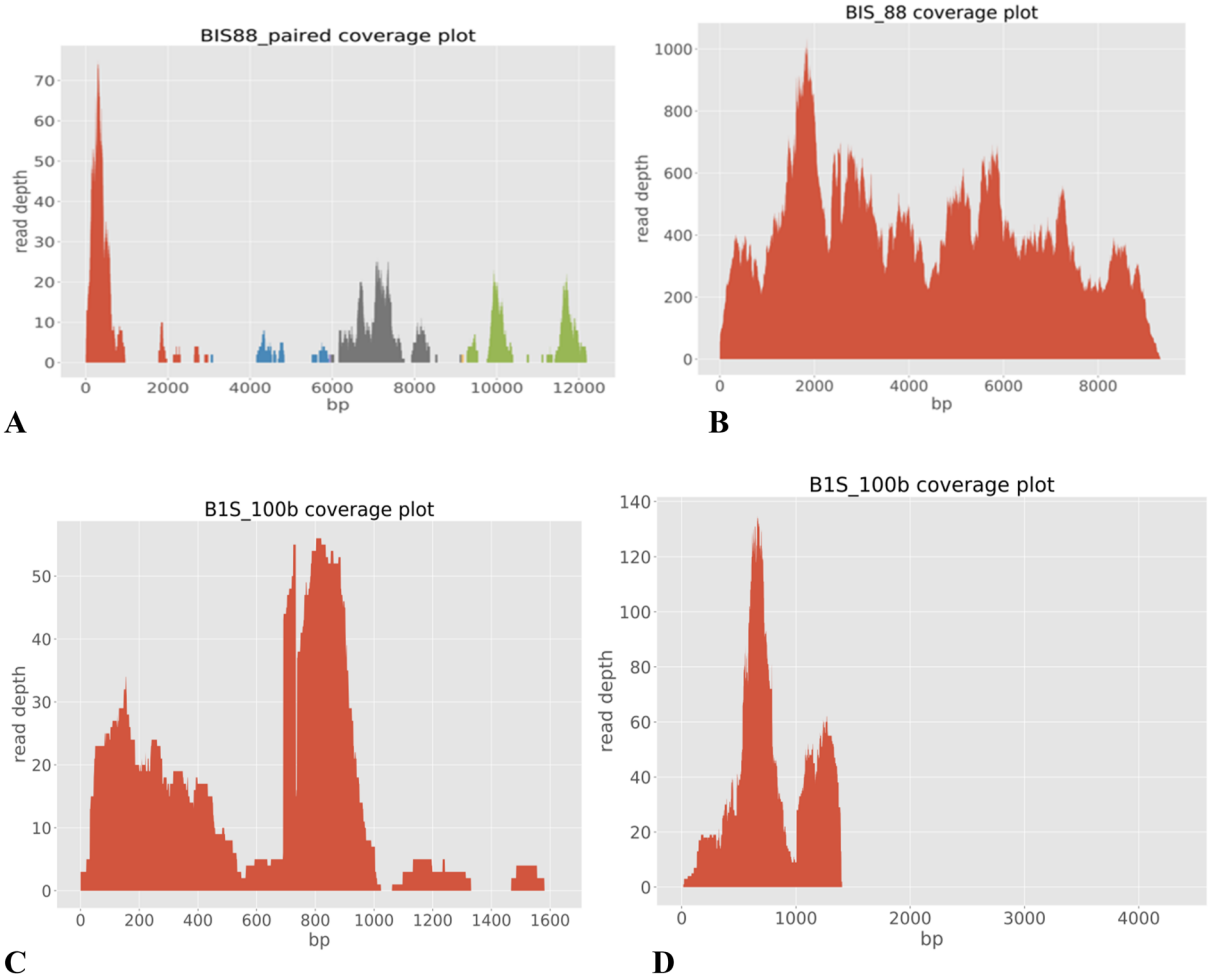
Coverage plot for (**A**) Fako virus segments, (**B**) Tesano Aedes virus, (**C**) Chaq like virus, (**D**) Verdadero virus. (**A**) Red = Segment 1, Blue = Segment 2, Grey = Segment 3 and Green = Segment 4.

Another Partivirus genome assembled in this study is the Verdadero virus (Table 4; Fig. 6). Verdadero virus was first isolated from *Aedes aegypti* colony from Poza Rica, Mexico. The name of the virus was derived from the Spanish word for “true” Verdadero^7^. There is no report on human infection by this virus. Generally, we did not observe any unique differences in viruses detected between male and female mosquito pools from the same collection site.

**Table 4:** Assembly and Genome characterization of members of *Iflaviridae*, *Partitiviridae*, *Flaviviridae* and *Totiviridae* described in this study.

Family	Genus (genome type)	Species	Isolate name	GenBank accession number	Genome length (bp)	Closest strain in GenBank	Identity (%)
Iflaviridae	Unclassified ssRNA ( +)	Tesano virus	B1S_88_T	OM522841	9310	LC496784.1 (16GH47)	93.30
			B1S_100c_T	OM522842	9339	LC496784.1 (16GH47)	97.17
			NB3_120_T	OM522845	9193	LC496784.1 (16GH47)	92.66
			NB3_150_T	OM522843	9313	LC496784.1 (16GH47)	98.15
Partitiviridae	Unclassified Partitivirus (dsRNA)	Chaq-like virus	B1S_100b_Cv	OM522892	1377	MT742176.1	98.77
		Verdadero virus	B1S_100b_V	OM522895	1313	MT742174.1	95.83
Flaviviridae	Unclassisfied Flavivirus ssRNA( +)	Cell fusing agent virus	B2S_50_CFAV	OM522840	4784	LR694076.1	97.79
Totiviridae	dsRNA	*Aedes aegypti* totivirus	B2S_50_AaT	OM522890	7974	MN053727.1	91.83
			B2S_51_AaT	OM522889	7557	MN053725.1	91.51
			NB3_58_AaT	OM522886	7735	MN053727.1	91.73
			B2S_83_AaT	OM522888	7677	MN053723.1	92.29
			B2S_100c_AaT	OM522887	7953	MN053723.1	92.81
			NB3_120_AaT	OM522885	4766	MN053726.1	92.78

Generally, reports on the effects of ISVs on pathogenic arbovirus transmissions are controversial and conflicting from various studies and in vivo*/*in vitro conditions.

Although ISVs cannot grow in mammalian cell lines, a study in Brazil isolated a novel insect-specific virus, Guapiaçu virus (GUAPV), from the plasma sample of a febrile person^9^. Furthermore, the discovery of ISVs within the families of pathogenic viruses provided insights into the evolution and adaptation of these groups of viruses^35^. For example, ISVs belonging to *Flaviviridae* and *Bunyaviridae* families are thought to be ancient viruses with distinct lineages that have evolved at the same time and diversified with their vector hosts^10,36,37^. Studies establishing vertical transmission^24,38^ and evidence of ISVs genomic sequence integration in the genome of insect vectors^39^ have supported the hypothesis. Against this background, many pathogenic arboviruses probably gained their dual-host range by an adaptive evolution process that conferred the ability to infect vertebrates to ISVs. There is limited information on the pathogenicity of ISVs to their insect host. Furthermore, ISVs were considered possible biological control agents for vectors and arboviruses of public health importance due to their characteristic lack of replication in mammalian cell lines. Given all these possible applications of ISVs, they are an exciting group of viruses for further investigations.

We could not detect YFV from this study, possibly because of our small amount of virus in the vectors that could not be properly amplified in the vectors. This could also be due to limitations in the protocol we used to construct the genomic libraries. Although we have used the same protocol to sequence and assemble YFV in human samples^13^ successfully. An approach targeted at enriching the Yellow fever virus in the mosquito vectors could have resulted in potential YFV assembly.

Identifying these diverse groups of viruses (ISVs) from our study is a first step in applying local genomics capacity within the country for a holistic approach to disease outbreaks. Further investigation of the pathogenic potential of these viruses, how they enhance/inhibit transmission of circulating arboviruses in Nigeria needs to be carried out.

## Methods

### Sample description

During active yellow fever virus outbreaks, mosquito samples were trapped across nine (9) states in Nigeria between 2017 and 2020 for metagenomic sequencing. Generally, three trapping methods were used in this study; egg, larval and adult collections. At each sampling point, the coordinates were taken using a global positioning system (GPS) gadget as described by^40^. Live adult mosquitoes collected in the field were immobilized using Ethyl Acetate. A total of 15 pools were mosquitoes collected at the immature stages and reared to the adult stage, while 11 pools were from mosquitoes collected directly as adults. These adult mosquitoes were unfed and hungry, as they were collected foraging for blood meal. All adult mosquitoes collected were morphologically identified to species level using keys of^41–43^ and then pooled based on species and sex into 50 mosquitoes per pool. They were then introduced into well-labelled Eppendorf tubes containing RNAlater. The tubes were stored in freezers for the duration of the surveillance to keep the samples genetically intact. Immature stages (pupae and larvae) are reared to adults before pooling.

A cohort of samples making 26 pools of 1,300 mosquitoes were sequenced. Twenty-five (25) pools were *Aedes *spp (*Aedes aegypti*, *Aedes albopictus*, *Aedes luteocephalus*, *Aedes simpsoni*, *Aedes simpsoni complex*) and one pool of *Anopheles coustani* (Supplementary Table S1). Mosquitoes were trapped by the National Arbovirus and Vectors Research Centre (NAVRC), Enugu, Enugu State, Nigeria.

### RNA extraction and metagenomics sequencing

A total of 1300 mosquitoes made into twenty-six (26) mosquito pools were sequenced based on the established unbiased protocol^14^. Briefly, Vector pools were initially homogenized in 1 ml of cooled Dulbecco's Modified Eagle Medium (DMEM) (composition-500 ml DMEM High Glucose (4.5 g/I) with l-Glutamine), 1 ml Penicillin–Streptomycin, 15 ml Fetal Calf Serum (FCS) 3% and 5 ml Amphotericin B) and 500 ml of Zirconia beads (Firma Biospec: 2.0 mm, Cat. No 1107912). The contents were macerated for 10 min on the Qiagen Tissuelyser LT followed by centrifuging at 4500×*g* for 15 min. According to the manufacturer's instructions, the supernatant was further used for RNA extraction using the QIAamp Viral RNA extraction kit (Qiagen, Hilden, Germany). Extracted RNA was turbo Dnased to remove contaminating DNA and cDNA synthesis was carried out according to the published protocol^13,14^. Sequencing libraries were made using the Illumina Nextera XT kit. Next generation sequencing using Illumina Miseq V2-500 cycle kit at 251 read length paired-end sequencing was carried out on Illumina Miseq at the African Centre of Excellence for Genomics of Infectious Diseases (ACEGID), Redeemer's University, Ede, Nigeria.

### Sequencing reads quality control

We added nuclease-free water (NTC) as a negative control for each sample preparation step. Before subsequent downstream analysis, we checked the water control for possible contaminations. All sequencing reads taxonomically classified as a given virus were first checked against possible reads from the NTC. We did a reference mapping of our reads against reference genomes on NCBI to rule out false positives. For this study, we only report viruses for which genomes are assembled**.**

### Bioinformatics analysis

Bioinformatics analysis was carried out on the sequence data generated to identify viruses present in the samples. First, we demultiplexed individual libraries and removed reads mapping to the human genome or other known technical contaminants (e.g., sequencing adapters). Raw reads from the sequencing machine were passed through Microsoft’s Premonition metagenomics pipeline (https://innovation.microsoft.com/en-us/premonition). We also carried out genome assembly of individual viruses detected using our publicly available viral-ngs^15,44^ and VGEA pipelines^45^.

Assembled genomes were annotated using ORFfinder (https://www.ncbi.nlm.nih.gov/orffinder/) with a 300-nt minimum length and the genome structure was annotated using NCBI Conserved Domain Database version 3.19 (expected value threshold of 1 × 10^–2^) with the NCBI viral genome database as references.

Multiple alignments of nucleotide sequences and deduced amino acids with those of reference strains from GenBank were analyzed using the MAFFT online version^46^. To determine the phylogenetic relationship of viruses detected from the mosquito pools, reference nucleotide sequences representing *Aedes aegypti* totivirus and Phasi Charoen-like phasivirus (strains with all three segments) were obtained from GenBank. The evolutionary history was inferred using the Maximum Likelihood method and Tamura-Nei model^47^ with 1000 bootstrap replicates. Bootstrap support is indicated by values on branches. Evolutionary analysis was conducted in MEGA X version 10.1.8^48^.

## Supplementary Information

Supplementary Information.

## Data Availability

All the sequences from this study are available on github: https://github.com/acegid/Vector_Genomics_Study and on NCBI with accession numbers OM522840- OM522901.
