# Supplementary material for: Metagenomic sequencing characterizes a wide diversity of viruses in field mosquito samples in Nigeria
Source: Sci Rep. 2022 May 10;12:7616. doi: 10.1038/s41598-022-11797-2 (PMC9090917; doi:10.1038/s41598-022-11797-2)
Supplement: Supplementary file 1 — Supplementary Information. [file 41598_2022_11797_MOESM1_ESM.docx]

**Supplementary Material**

**Metagenomic sequencing characterizes a wide diversity of viruses in field mosquito samples in Nigeria**

​​Judith U. Oguzie^1,2^, Udoka C. Nwangwu^3^, Paul E Oluniyi^1,2^, Testimony J. Olumade^1,2^, Uwem E. George^1,2^, Akano Kazeem^1,2^, Bolajoko E. Bankole^1,2^, Farida O. Brimmo^1^, Chukwuemeka C. Asadu^3^, Okechukwu C. Chukwuekezie^3^, Josephine C. Ochu^3^, Catherine O. Makwe^4^, Festus A. Dogunro^3^, Cosmas O. Onwude^3^, William E. Nwachukwu^5^, Ebuka K. Ezihe^3^, Gilkenny K. Okonkwo^4^, Ndubuisi E. Umazi^4^, Jacob Maikere^4^, Nneka O. Agashi^3^, Emelda I. Eloy^3^, Stephen O. Anokwu^3^, Angela I. Okoronkwo^3^, Ebuka M. Nwosu^3^, Sandra O. Etiki^3^, Ifeoma M. Ngwu^3^, Chikwe Ihekweazu^5^, Onikepe A. Folarin^1,2^, Isaac O.O Komolafe^1,2^, Christian T. Happi^1,2*^

1. African Centre of Excellence for Genomics of Infectious Diseases (ACEGID), Redeemer’s University, Ede, Osun State, Nigeria.
2. Department of Biological Sciences, Faculty of Natural Sciences, Redeemer’s University, Ede, Osun State, Nigeria.
3. National Arbovirus and Vectors Research Centre (NAVRC), Enugu, Enugu State, Nigeria
4. Médecins Sans Frontières (MSF Belgium).
5. Nigeria Center for Disease Control, Abuja, Nigeria.

*Correspondences: Professor Christian T. Happi (email: [happic@run.edu.ng](mailto:happic@run.edu.ng); Tel: +234-8023383684)

Supplementary Table S1: Metadata

| ID | STATE | SPECIES | SEX | TOTAL SEQUENCING READS | MEAN SEQUENCE QUALITY (PHRED SCORE) |
| --- | --- | --- | --- | --- | --- |
| B1S_20 | Ebonyi | *Ae. simpsoni complex* | female | 692232 | 36 |
| B1S_45 | Anambra | *Ae. albopictus* | female | 2503602 | 37 |
| B1S_59 | Edo | *Ae. aegypti* | female | 3469076 | 37 |
| B1S_70 | Osun | *Ae. albopictus* | female | 2724416 | 37 |
| B1S_88 | Edo | *Ae. aegypti* | female | 849566 | 37 |
| B1S_100b | Kwara | *Ae. aegypti* | female | 1498252 | 37 |
| B1S_100c | Kwara | *Ae. aegypti* | female | 2755048 | 35 |
| B1S_101 | Kwara | *Ae. aegypti* | female | 1700618 | 35 |
| B1S_102A | Kwara | *Ae. Aegypti* | female | 1296946 | 35 |
| B2S_15 | Ebonyi | *Ae. albopictus* | female | 993776 | 36 |
| B2S_48 | Ebonyi | *Ae. albopictus* | female | 2892896 | 35 |
| B2S_50 | Ebonyi | *Ae. albopictus* | female | 1717588 | 37 |
| B2S_51 | Ebonyi | *Ae. albopictus* | female | 2308992 | 37 |
| B2S_69 | Ebonyi | *Ae. albopictus* | female | 876344 | 37 |
| B2S_73 | Ebonyi | *Ae. aegypti* | female | 1715760 | 36 |
| B2S_75 | Ebonyi | *Ae. albopictus* | female | 1221702 | 36 |
| B2S_83 | Ebonyi | *Ae. albopictus* | female | 2796030 | 33 |
| B2S_91 | Ebonyi | *Ae. aegypti* | female | 1012258 | 25 |
| NB3_58 | Ebonyi | *Ae. aegypti* | male | 449324 | 35 |
| NB3_99 | Nassarawa | *Ae. luteocephalus* | female | 1414536 | 35 |
| NB3_120 | Nassarawa | *Ae. aegypti* | male | 445198 | 35 |
| NB3_122 | Ebonyi | *Ae. albopictus* | male | 247396 | 34 |
| NB3_126 | Ebonyi | *Ae. aegypti* | female | 1484712 | 36 |
| NB3_146 | Niger | *Anopheles coustani* | female | 872232 | 35 |
| NB3_150 | Benue | *Ae. aegypti* | female | 941410 | 35 |
| NB3_173 | Enugu | *Ae. simpsoni complex* | female | 1364862 | 33 |


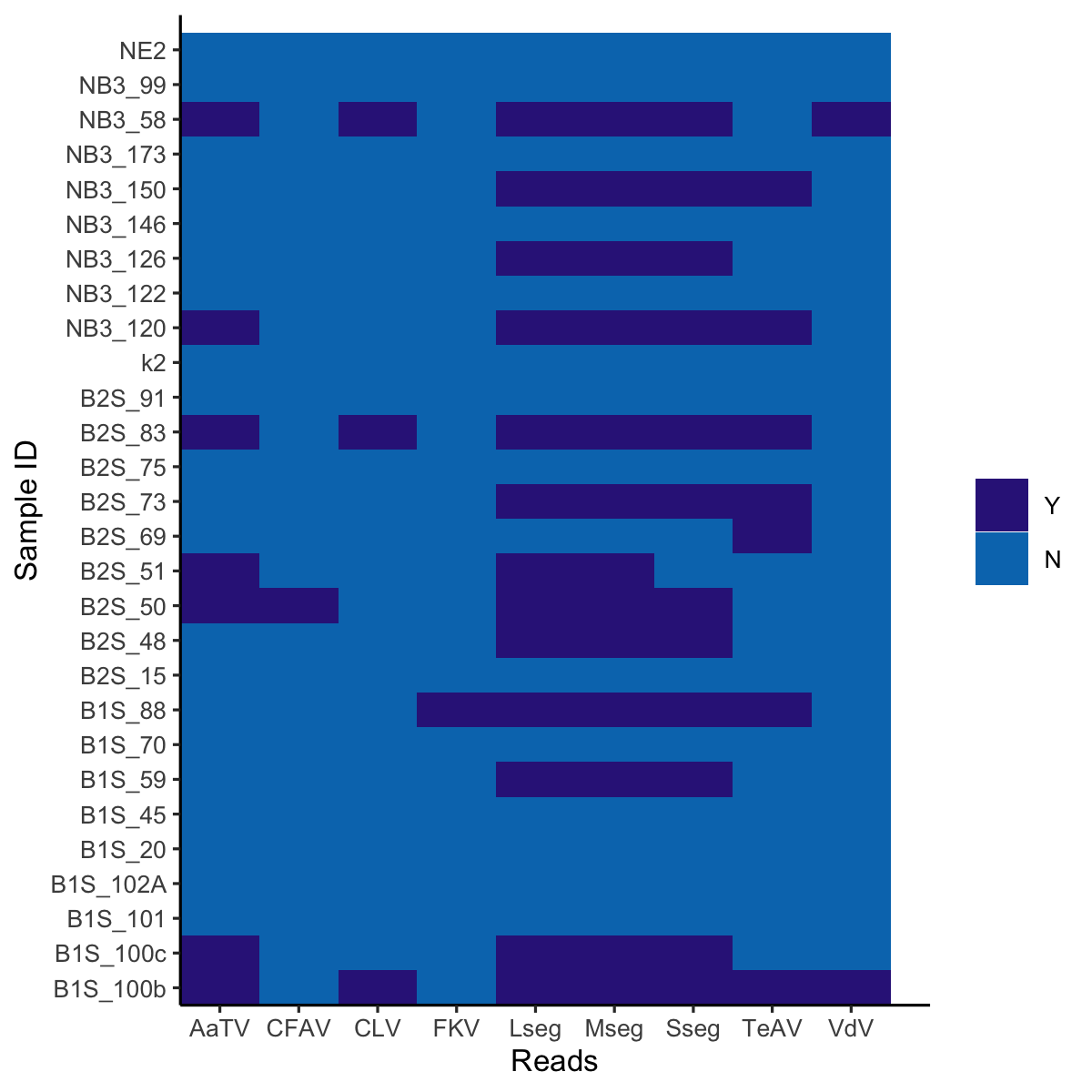


Supplementary Figure S1: Heatmap of Assembled Viruses.

Legend: AaTV =Aedes aegypti totivirus, CLV= Chaq like-virus, CFAV= cell fusing virus, FKV= Fako virus, LSeg = L segment of Phasi Charoen like phasivirus, MSeg = M segment of Phasi Charoen like phasivirus, SSeg = S segment of Phasi Charoen like phasivirus, TeAV= Tesano Aedes virus, VdV= Verdadero virus.
